# Effects of the COVID-19 Pandemic on Place in End-of-Life Care: Insights From a Homeless Mobile Palliative Care Team

**DOI:** 10.1093/geroni/igab046.1457

**Published:** 2021-12-17

**Authors:** Ian Johnson

**Affiliations:** University of Washington, SEATTLE, Washington, United States

## Abstract

The effects of the COVID-19 pandemic on both those experiencing homelessness (Tsai & Wilson, 2020) and those with life-limiting illnesses (Abbott et al., 2020) is of great public health concern. This presentation details the findings from an organizational case study (Yin, 2014) aimed at investigating COVID-related changes to the service environments in which unhoused palliative care patients receive care. Through ethnographic field observation (Phillippi & Lauderdale, 2017) and interviews with a homeless palliative care team and their community partners (Turner, 2005), findings included 1) decreased staff capacity due to de-congregated care; 2) efforts to extend care in community settings due to relocation barriers; 3) conflict between reducing viral risk and honoring unique population needs; and 4) provider perceptions of COVID-19 as an “equalizer.” Findings illustrate the impact of emergency response within housing and healthcare systems on unhoused patients’ care and offer potential pathways to quality end-of-life care for homeless populations.

